# Clustering exact matches of pairwise sequence alignments by weighted linear regression

**DOI:** 10.1186/1471-2105-9-102

**Published:** 2008-02-18

**Authors:** Alvaro J González, Li Liao

**Affiliations:** 1 Laboratory of Bioinformatics, Computer and Information Sciences Department, University of Delaware, 421 Smith Hall, Newark, DE 19716, USA

## Abstract

**Background:**

At intermediate stages of genome assembly projects, when a number of contigs have been generated and their validity needs to be verified, it is desirable to align these contigs to a reference genome when it is available. The interest is not to analyze a detailed alignment between a contig and the reference genome at the base level, but rather to have a rough estimate of where the contig aligns to the reference genome, specifically, by identifying the starting and ending positions of such a region. This information is very useful in ordering the contigs, facilitating post-assembly analysis such as gap closure and resolving repeats. There exist programs, such as BLAST and MUMmer, that can quickly align and identify high similarity segments between two sequences, which, when seen in a dot plot, tend to agglomerate along a diagonal but can also be disrupted by gaps or shifted away from the main diagonal due to mismatches between the contig and the reference. It is a tedious and practically impossible task to visually inspect the dot plot to identify the regions covered by a large number of contigs from sequence assembly projects. A forced global alignment between a contig and the reference is not only time consuming but often meaningless.

**Results:**

We have developed an algorithm that uses the coordinates of all the exact matches or high similarity local alignments, clusters them with respect to the main diagonal in the dot plot using a weighted linear regression technique, and identifies the starting and ending coordinates of the region of interest.

**Conclusion:**

This algorithm complements existing pairwise sequence alignment packages by replacing the time-consuming seed extension phase with a weighted linear regression for the alignment seeds. It was experimentally shown that the gain in execution time can be outstanding without compromising the accuracy. This method should be of great utility to sequence assembly and genome comparison projects.

## Background

Genome sequencing is the process to determine the exact sequential order in which a target organism's DNA is made up by the building blocks, called bases and abbreviated A, C, T, G. This process is carried out in two phases. The first phase is shotgun sequencing phase where the DNA molecule of the organism is randomly sheared into a large number of small fragments and the ends of the fragments are read base by base using a chemical procedure introduced by Sanger [1]. The second phase is assembly phase where the resulting sequences of these fragments are put together by a program (called the assembler) that attempts to join these fragments by their overlapping sequences in order to restore the entire target genome. In an ideal case, the assembly phase would yield one single long string in the alphabet {A, C, T, G}, but this is far from being the reality, even when the sequencing phase is careful enough to cover the whole genome by several fold factors. Typically two issues prevent this ideal situation from happening: non-random shearing and repeats. Non-random shearing occurs because all organisms have in their DNA sequence regions that are more difficult to be sequenced than others, which in some cases transcends into gaps that are not sequenced at all. On the other hand, repeats are sequences extracted from different regions of the genome that have a near identical primary structure. When fragments from these regions are sequenced, the assembler is not able to differentiate them, and most likely joins them as if they came from a common region, a mistake that is hard to avoid. As a consequence of these two challenging situations, the assembler cannot piece these fragments to restore the entire target genome as a single sequence; instead, it assembles the fragments into some long contiguous sequences, called contigs. These contigs are separated by gaps, namely, they do not share overlapping flanks, which are necessary in order to further piece these contigs together. Even the relative order of these contigs in the genome is not known. The next stage in the assembly phase is to identify the relative order.

A typical technique for ordering the contigs is called scaffolding which uses paired reads. It is worth noting that the scaffolding by paired reads does not guarantee a complete ordering (see [2] for details). Once the contigs are ordered, the gaps that separate them can be identified and associated to the fragments (clones) that are not sequenced properly in the shotgun sequencing phase. Gap closure will involve resequencing these fragments and/or acquiring new fragments of DNA in the corresponding regions. Scaffolding and gap closure are tedious and laborious processes, hard to be automated. However, when the genome of a closely related organism has already been sequenced, it can then be used as a reference, *i.e*., the contigs of the target genome can be matched against the reference genome. The alignments of these contigs onto the reference genome can potentially produce an informative overall picture of the ordering of the contigs, and therefore greatly facilitate the scaffolding and gap closure. Using reference genomes in sequence assembly has become increasingly feasible as more genomes have been sequenced and therefore become available as potential references.

Many software packages are available for doing pairwise sequence alignments, including Fasta [3], Blast [4], BLAT [5] and MUMmer [6]. Fasta and Blast compare two sequences to discover regions of high similarity while allowing insertions, deletions and point mutations. BLAT is inspired in Blast, but introduces several changes to speed up the alignment process. MUMmer looks for the kinds of large-scale changes that can be discovered in whole-genome comparisons. Nonetheless all of these algorithms operate in a similar way; they initially find local exact matches (called "seeds") between the two sequences, and then the seeds are extended to fill the gaps using constrained versions of the Smith-Waterman algorithm [7].

In assembly projects, when a set of contigs is compared against a reference genome, the interest is to find out whether each contig is potentially an actual segment of the target genome and to establish the positional order of all the contigs in the target genome. For these purposes, a detailed base to base alignment is not needed. It suffices to find only exact match regions, therefore the seed extension procedure can be skipped. MUMmer, which uses a suffix tree approach to directly find exact matches between the two sequences, is a tool best suited for this task due to its efficiency in both time and space, as a result of using the suffix tree data structure.

**Figure 1:**
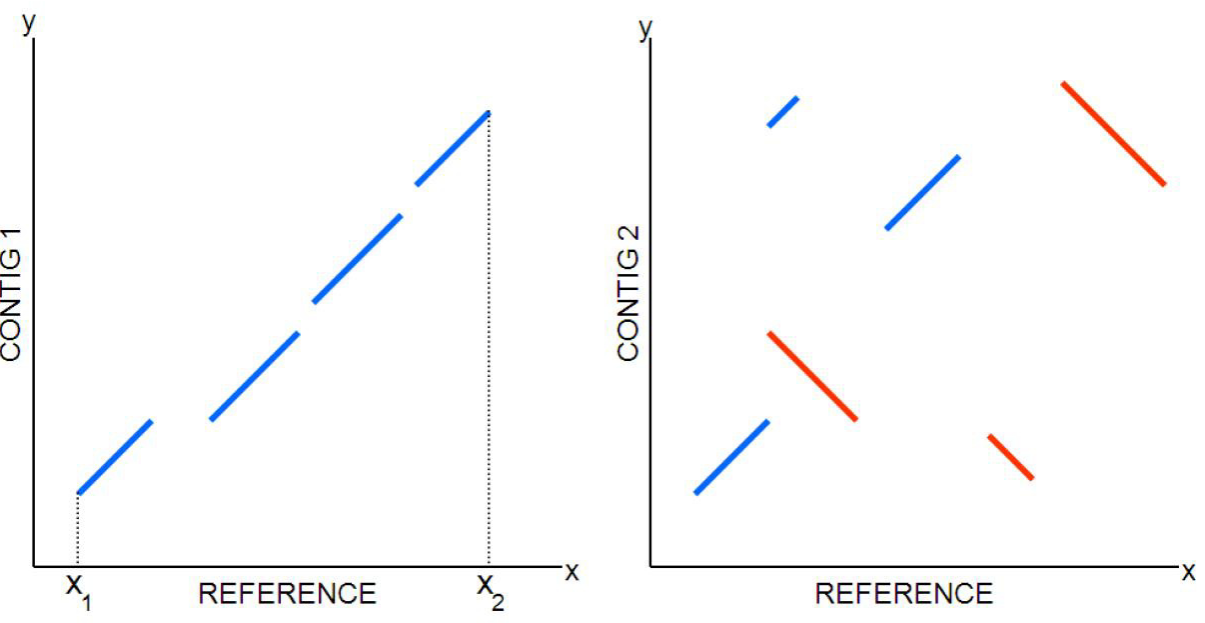
**The dot plot of the alignment of two different contigs to a reference sequence**. The dot plot of two different contigs as they are aligned to a reference genome. Contig 1 (left) strongly aligns to the region between *x*_1 _and *x*_2_, but Contig 2 (right) does not align as a whole.

The exact matches that MUMmer extracts can be represented in a dot plot as shown in Figure 1. When the aligned contig is a good prospect, *i.e*. the contig accurately reconstructs a region in the target genome that also exists in the reference, the seeds of the alignment should be approximately collinear (Figure 1, left). On the other hand, the seed alignments, as shown in Figure 1 (right), can be skewed and shifted away from the main diagonal, indicating some significant mismatches between the contig and the reference. These mismatches may represent genuine genetic variances, such as translocation, inversion and insertion between the target and reference genomes, but they can also be due to sequence errors in the contig arising in misassembly. In both cases, the alignment provides less useful information to the assembly process. Also, since there is no reliable way to differentiate these two cases, these contigs should be flagged for further analysis.

In many cases, a visual assessment of the dot plot of the seeds of an alignment is enough to judge the reliability of a contig. However, more exact numerical figures are often needed to validate a contig. Besides, it is common to have sets of thousands (and even hundreds of thousands) of contigs to be matched against a reference, in which case the graphical method is ruled out. In this work, we propose a simple yet powerful algorithm that takes as input the set of exact matches (seed alignments) between a contig and a reference genome, and produces as output the starting and ending coordinates of the most likely global alignment that exists between the two sequences. In Figure 1 (left), the output would be (*x*_1_, *x*_2_). The method also assigns each alignment a score to assess its goodness so that non reliable contigs can be easily flagged for further analysis or even discarded directly. The method is based on clustering the seed alignments using a weighted linear regression, which is presented in the next two sections.

## Methods

### Motivation for a Weighted Pairwise Linear Regression

**Figure 2:**
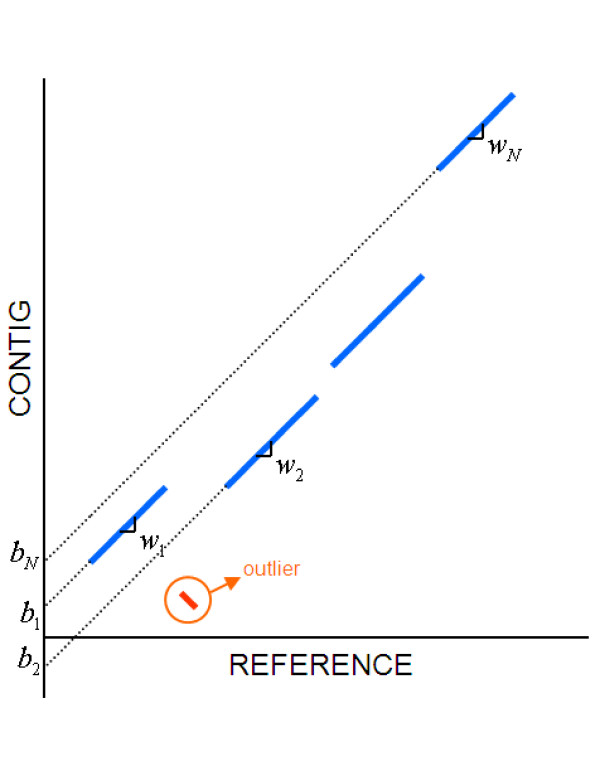
**Set of exact matches corresponding to a contig that aligns to a region in a reference sequence**. Each exact match (seed) in an alignment can be characterized by its slope *w*_*i *_and its *y*-intercept *b*_*i*_.

Assume we run MUMmer to align a given contig to a reference genome. MUMmer can be tuned to find only exact matches and filter out those matches that do not occur in the same order in both sequences. If the contig has a strong alignment to the reference, the seeds should line up in a collinear manner in the dot plot like what is shown in Figure 2. Each seed is characterized by its slope *w*_*i *_(since these seeds are for exact matches, slopes can only be -1 or 1) and a *y*-intercept *b*_*i*_. Our goal is to identify a strong alignment between the contig and reference, cluster the seeds that would belong to such an alignment, and filter out the "outliers" (those matches that are far away from the diagonal region that characterizes the global alignment, see Figure 2). Therefore, we want to find a straight line with slope $\bar{w}$ and *y*-intercept $\bar{b}$ that can serve as a collinear axis for these segments representing the seeds, in such a way that the seeds not in line and/or far way from the axis can be identified as outliers and be excluded. Once the axis is determined and segments are aligned to it, the boundary coordinates of the global alignment can be easily found.

One way to approach this problem is by standard linear regression for all the starting and ending points of the seeds. However this solution does not take into account the fact that we are trying to align segments, not individual points. So we propose a slightly different modification of linear regression that not only accounts for the pairing of points, *i.e*., as both ends of a segment, but also weighs in the length of segments. In order to find $\bar{w}$ and $\bar{b}$, we minimize the following metric:

(1) $$C=\sum_{i=1}^{N}l_{i}\cdot \left({\left(w_{i}-\bar{w}\right)}^{2}+{\left(b_{i}-\bar{b}\right)}^{2}\right)$$

where *l*_*i *_is the length (in bases) of each match. It is reasonable to assign this weight to each seed, since the longer a match is, the more significant weight it should be given in determining the collinear axis.

Differentiating the metric with respect to $\bar{w}$ and $\bar{b}$ we get:

(2) $$\frac{dC}{d\bar{w}}=\frac{dC}{d\bar{b}}=-2\cdot \sum_{i=1}^{N}l_{i}\cdot \left(\left(w_{i}-\bar{w}\right)+\left(b_{i}-\bar{b}\right)\right)$$

which is made equal to zero to solve for $\bar{w}$ and $\bar{b}$, hence we finally obtain:

(3) $$\begin{array}{lll} \bar{w}=\frac{\sum_{i=1}^{N}l_{i}w_{i}}{\sum_{i=1}^{N}l_{i}} & \text{and} & \bar{b}=\frac{\sum_{i=1}^{N}l_{i}b_{i}}{\sum_{i=1}^{N}l_{i}}. \end{array}$$

Eq. (3) suggests that the optimal $\bar{w}$ and $\bar{b}$ for this weighted pairwise linear regression can be obtained as arithmetic average over the slopes and the *y*-intercepts, respectively. It is interesting to note that this solution is surprisingly simpler than that of a standard linear regression, which involves computation equivalent to matrix inversion. The implementation of Eq. (3) is straightforward, and the results have an accuracy of about 70% as compared to a large set of human curated alignments.

This initial idea served as an inspiration for an improved algorithm, more adapted to the nature of the problem. Because of the very nature of linear regression, be it a standard one or a modified one like ours, the solution is necessarily a kind of compromise, which means that it can be skewed if many small scattered outliers exist. To mitigate such a problem, a further modification to the algorithm is presented in the next section.

### The modified algorithm

Following is a description in pseudo-code of the final algorithm that we developed to process the seed alignments (*i.e*., matched segments) between a contig and a reference sequence. Each seed alignment is described by a 4-tuple (*s*_*x*_, *s*_*y*_, *e*_*x*_, *e*_*y*_), where *s*_*x *_and *e*_*x *_are the starting and ending coordinates on the reference sequence, and *s*_*y *_and *e*_*y *_are the starting and ending coordinates on the contig sequence. As mentioned before, the goal is to find the boundary coordinates (*x*_1_, *x*_2_) of the region in the reference (as x-axis) that more strongly aligns to the contig (as y-axis), as shown in Figure 1 (left).

Input:

*S *= {*S*_*i*_, *i *= 1, 2, ..., *N*}, a set of *N *matched segments, each is a 4-tuple (*s*_*x*_, *s*_*y*_, *e*_*x*_, *e*_*y*_); and *v*, a window size given as a percentage of the contig length.

Algorithm:

1. Compute the accumulated lengths *L*_*f *_and *L*_*r *_for matched segments on forward strand and on reverse strand respectively.

   **FOR EACH ***S*_*i *_∈ *S*

   **IF **(slope(*S*_*i*_) > 0)

   **THEN ***L*_*f *_+= ||*S*_*i*_||

   **ELSE ***L*_*r *_+= ||*S*_*i*_||.

2. Decide on the dominant strand by the maximum of *L*_*f *_and *L*_*r*_.

   **IF **(*L*_*f *_> *L*_*r*_)

   **THEN ***DS *= +1

   **ELSE ***DS *= -1.

3. Project the segments on the dominant strand onto the *Y*-axis and assign them each a weight *λ *defined as the total length of segments whose *y*-intercept is within a window of size 2*v*.

   **FOR EACH ***S*_*i *_∈ *S*

      **IF **(slope(*S*_*i*_) = *DS*)

      **THEN ***λ*_*i *_= ∑_*j *_||*S*_*j*_||, for all segments *S*_*j *_whose *y*-intercept *b*_*j *_satisfies *b*_*i *_- *v *≤ *b*_*j *_≤ *b*_*i *_+ *v*.

4. Find the segment, indexed as i*, that has the maximum weight. That is to say, segment *S*_i* _has most long segments within the window of the given size centering at it.

i* = arg max_*i*_(*λ*_*i*_) and b* = *b*_i*_.

5. Use the segment *S*_i* _as a centroid to cluster all segments whose *y*-intercept falls within the window of size 2*v *centering at b*. Find the indexes j* and k* for the two boundary segments of the region where the contig and the reference are strongly aligned.

   $j^{*}=\arg\min_{S_{j}}\left(s_{x}^{2}+s_{y}^{2}\right)$, for all segments *S*_*j *_whose *y*-intercept *b*_*j *_∈ [b* - *v*, b* + *v*];

   $k^{*}=\arg\max_{S_{k}}\left(e_{x}^{2}+e_{y}^{2}\right)$, for all segments *S*_*k *_whose *y*-intercept *b*_*k *_∈ [b* - *v*, b* + *v*].

Output:

   (*x*_1_, *x*_2_) = (*s*_*x*_, *e*_*x*_), where *s*_*x *_is the starting coordinate of segment *S*_j* _and *e*_*x *_is the ending coordinate of segment *S*_k*_, all with respect to the reference sequence.

   (*y*_1_, *y*_2_) = (*s*_*y*_, *e*_*y*_), where *s*_*y *_is the starting coordinate of segment *S*_j* _and *e*_*y *_is the ending coordinate of segment *S*_k*_, all with respect to the contig sequence.

It is noted that the length of the alignment |*y*_2 _- *y*_1_| can be compared with the contig length, and a significant difference between the two can be used to flag problematic contigs for further analysis.

Specifically, each contig is assigned a score based on its alignment with the reference defined as follows:

(4) $$\begin{array}{lll} Score(S) & = & \frac{\min(|x_{1}-x_{2}|,|y_{1}-y_{2}|)}{\max(|x_{1}-x_{2}|,|y_{1}-y_{2}|)}\\ & + & \frac{\left(\sum_{i=1}^{N}\left\|S_{i}\right\|\right)}{L}\\ & + & \frac{\max\left(L_{f},L_{r}\right)}{\left(\sum_{i=1}^{N}\left\|S_{i}\right\|\right)}\\ & + & \frac{\sum_{j\in \zeta }\left\|S_{j}\right\|}{\max\left(L_{f},L_{r}\right)}. \end{array}$$

The right hand side of this equation has four terms. The first term is a ratio between the lengths of the estimated alignment on the contig and the reference, with the denominator always being the bigger of the two. This ratio therefore gives a sense of insertions and deletions within the estimated alignment; a perfect alignment without any insertion and deletion receives a value of 1, and the lower value implies more deletions and insertions. The second term is a ratio between the accumulated length of seeds and the contig length. The third term is a ratio of the accumulated length of seeds in the dominant strand over the total seeds length. In the fourth term, *ζ *is the subset of seeds that were clustered, namely, *ζ *consists of all segments *S*_*j *_whose *y*-intercept *b*_*j *_∈ [b* - *v*, b* + *v*], as was defined in step 5 of the algorithm. Therefore, the fourth term is the ratio of the total length of seeds in the dominant strand and clustered over the total length of seeds in the dominant strand. All these ratios are valued theoretically in the range [0, 1], with 1 corresponding to a perfect alignment between contig and reference. For convenience, the score is then scaled to the range [0, 100] by multiplying the right hand side of Eq. 4 by 25.

As for the time complexity, after the *y*-intercepts are sorted, all steps except step 3 run in time obviously as a linear function of *N*, which is the number of matched segments. The complexity for step 3 is also linear with the following refined implementation. By keeping two pointers, one to the low intercept value and one to the high value, the sum of lengths can be computed for the next match by advancing both pointers, subtracting out low values that are now too low and adding in new high values. Both pointers scan linearly through the data for total linear time. It is noted that, even with the less refined implementation, which can be *O*(*N*^2^) as suggested in the pseudo code, step 3 runs effectively in linear time when the window size is kept small.

**Figure 3:**
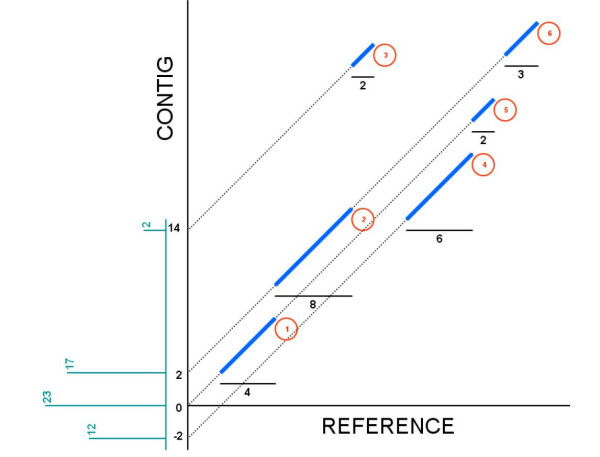
**Matches in the forward strand, and their corresponding weights according to the proposed algorithm**. Matches in the forward strand, their *y*-intercepts and the weight (length) of each match. The algorithm maximizes the discrete plot shown in green.

Now we present a simple example to help illustrate the mechanics of the algorithm. Suppose MUMmer found six exact matches in the forward strand, as shown in Figure 3, where each seed has been numbered (in circled red numbers). The length of each seed in the reference sequence is also shown (black horizontal lines). The algorithm should be able to cluster all matches but match No. 3 (an outlier).

Assume a window of size 4 for this example. Forward strand is chosen as the predominant strand of the alignment. Then the *y*-intercept and the weight vectors are found to be $\vec{b}$ = (14, 2, 0, -2) and $\vec{\lambda }$ = (2, 17, 23, 12). In Figure 3, a discrete plot with $\vec{b}$ in the horizontal and $\vec{w}$ in the vertical axis is shown in green. For instance, for *y*-intercept 14 only match No. 3 is within its window range, hence a weight of 2 was assigned. For *y*-intercept 2, matches No. 1, 2, 5 and 6 are inside its window range, then a weight 4 + 8 + 2 + 3 = 17 was assigned. By finding the maximum of $\vec{\lambda }$, which is 23, we conclude that $\hat{b}$ = 0. Then all the matches whose *y*-intercepts are inside the window range of $\hat{b}$ are clustered, and the ones outside this region are declared outliers.

## Results and Discussion

We present in this section alignment results from real assembly data. In our attempt to assemble a region of the genome of several rice species, sets of contigs of varied cardinality and average length are produced and aligned to an available reference genome, namely the sequence of *O. sativa *var. *japonica*. Our algorithm was implemented in a perl script that takes as input the alignments generated from MUMmer. The pipeline consists of two steps: running MUMmer to find the seed alignments, and clustering these seed alignments by the weighted linear regression method to identify the mapping region. MUMmer's parameters can be tuned up such that only a consistent sequence of seeds is found, avoiding the extension phase in MUMmer that tries to fill the gaps between seeds using Smith-Waterman algorithm (this phase is the most time consuming part of the whole MUMmer pipeline). Our perl script parses MUMmer output file, and for each contig in the set it produces the two boundary coordinates of the region in the reference where the contig most likely aligns. We also give a score (from 0 to 100) to each projected mapping coverage based on the statistics and aggregate length of the set of seeds that compose each alignment as compared to the actual contig's length.

On the other hand, it is also possible to force MUMmer to make aggressive efforts to fill all the gaps and give as a result one extended alignment between the contig and the reference. Notice this is the goal of the weighted linear regression algorithm, but as will be seen, MUMmer takes much longer while trying to align the gapped regions base by base.

The first thing to decide before running the algorithm is the window size. When long contigs are being aligned, a considerable number of insertions, deletions and miss-matches are expected, so the width of the diagonal region of the clustered matches should be widen. It is for this reason that we choose the size of the window to be proportional to the contig's length. This size is input to the program as a percentage that can go from 1% (the window size is 1/100 of the contig's length) to 100% (the window size is the whole contig's length).

**Figure 4:**
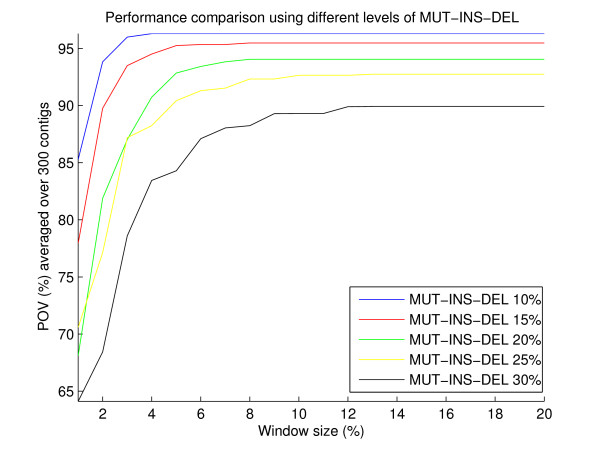
**Performance curve with respect to window size**. Performance curve with respect to window size with different levels of Mut-Ins-Del.

We tested the performance of the algorithm as a function of window size (seen as percentage of contig's length) and the results were plotted in Figure 4. To evaluate the window size effect, we created artificial contigs picked randomly from regions in the reference chromosome, which itself is a randomly generated sequence of about 35 Mb long. Use of this long artificial chromosome allows for sufficient sampling and low rate of repeats. A total of 300 contigs were produced and were grouped based on their lengths into three subsets: 1 *kb*, 10 *kb*, and 100 *kb*, with 100 contigs in each subset. Each contig was sprinkled randomly with regions of mutations, insertions and deletions according to a given percentage of the contig length. For instance, a Mut-Ins-Del percentage of 10% indicates that the initial contig was first contaminated randomly with regions of mutations whose accumulated length amounts to 10% the length of the contig. Then regions of insertions under the same principle were introduced, and likewise with deletion regions. Since the location of the contig in the reference is known a priori, after these contigs are aligned to the reference using MUMmer and the predominant alignment region is predicted using our proposed algorithm, we can measure the percentage of the overlap (POV) between the two regions (predicted and real). We did so for all window sizes from 1% to 20%, as shown in Figure 4. Different levels of Mut-Ins-Del were tested.

As expected, when mutations, insertions and deletions are low, a small window size is sufficient to maximize the POV between real and predicted regions, and as they are increased, longer windows are needed to accurately cluster all the exact matches of the alignment. Notice that this window size is desired to remain as low as possible to improve the speed of the algorithm. Our analysis suggests that a window size of 12% works well in all cases.

**Figure 5:**
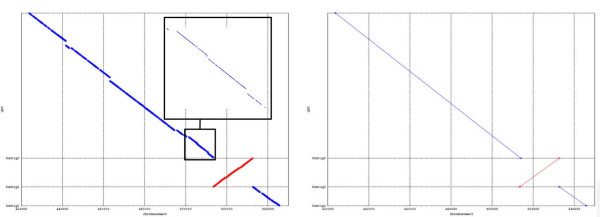
**Comparison of dot plots generated by MUMmer**. Dot plot after aligning a set of three contigs against chromosome 3 of *japonica *rice. In the left, MUMmer was tuned up to find only exact matches between the contigs and the chromosome. A zoom in of certain region of the dot plot is shown. In the right MUMmer was forced to fill all gaps between seeds to yield one solid alignment per contig.

Table 1 shows the execution time of MUMmer when it is forced to find one solid alignment per contig as compared to the execution time of MUMmer (tuned up to find only exact matches). For comparison, the last column shows the time our algorithm takes clustering the seeds and calculating the boundary coordinates of the alignment. Seven sets of contigs are analyzed, and the number of contigs along with the average contig length is shown for each set. Figure 5 shows the seeds (left) and the solid alignment (right) of the contigs from Set 2 (second row in Table 1). Although outliers (matches outside the diagonal region) are not seen, they do exist, but the length of the reference chromosome (around 35 Mb) makes it prohibitive to show the complete picture of the alignment. Table 1 shows that the weighted linear regression was much faster in all the sets, being 145 and 2 times faster in the best (Set 2) and the worst (Set 7) cases, respectively. The weighted linear regression script accurately found the boundary coordinates of the alignment for 100% of the contigs shown in Table 1 verified visually by analyzing the dot plot of all the alignments. It is worth noting that, although MUMmer has been used in this study, other software packages like BLAT can be used in its place in generating the seed alignments. In fact, BLAT runs faster than MUMmer in our testing for generating the seed alignments, though it does not seem to produce long extension as MUMmer does.

**Table 1:** Comparison of results.

	No. of contigs	Average contig length	Time in MUMmer	Time using linear regression
Set 1	2	60848	102.003 s	0.922 + 0.037 = 0.959 s
Set 2	3	43489	124.064 s	0.823 + 0.034 = 0.857 s
Set 3	3	41227	108.776 s	0.889 + 0.034 = 0.923 s
Set 4	9	14389	39.470 s	0.881 + 0.047 = 0.928 s
Set 5	44	2621	5.182 s	0.881 + 0.127 = 1.008 s
Set 6	36	2210	2.963 s	0.884 + 0.102 = 0.986 s
Set 7	18	1810	1.772 s	0.861 + 0.053 = 0.914 s

Comparing alignment of several sets of contigs (assembled from a BAC in *O. glaberrima*) to a reference chromosome (in *O. sativa*) using only MUMmer and using MUMmer with weighted linear regression. The time using linear regression is shown as the sum between the time MUMmer takes to find the set of seeds plus the execution time of the weighted linear regression algorithm. (See the text for detailed discussion).

## Conclusion

We presented a method to cluster exact matches of pairwise sequence alignments by weighted linear regression which can be of great utility for genome assembly projects. This algorithm complements existing pairwise sequence alignment packages by avoiding their seed extension phase and replacing it with a weighted linear regression in the seeds of the alignment. It was experimentally shown that the gain in execution time can be outstanding without necessarily compromising the accuracy.

## Availability

The perl program is available at .

## Authors' contributions

LL conceived the idea of alignments clustering, designed and prototyped the weighted Pairwise linear regression. AJG designed and implemented the modified algorithm and the experiments. AJG drafted the manuscript, and LL provided critical edits and important intellectual content. Both authors have read and approved the final version of this manuscript.
